# An Intronic SINE Insertion in *FAM161A* that Causes Exon-Skipping Is Associated with Progressive Retinal Atrophy in Tibetan Spaniels and Tibetan Terriers

**DOI:** 10.1371/journal.pone.0093990

**Published:** 2014-04-04

**Authors:** Louise M. Downs, Cathryn S. Mellersh

**Affiliations:** Kennel Club Genetics Centre, Animal Health Trust, Newmarket, United Kingdom; University of Sydney, Australia

## Abstract

Progressive retinal atrophy (PRA) in dogs is characterised by the degeneration of the photoreceptor cells of the retina, resulting in vision loss and eventually complete blindness. The condition affects more than 100 dog breeds and is known to be genetically heterogeneous between breeds. Around 19 mutations have now been identified that are associated with PRA in around 49 breeds, but for the majority of breeds the mutation(s) responsible have yet to be identified. Using genome-wide association with 22 Tibetan Spaniel PRA cases and 10 controls, we identified a novel PRA locus, PRA3, on CFA10 (p_raw_ = 2.01×10^−5^, p_genome_ = 0.014), where a 3.8 Mb region was homozygous within 12 cases. Using targeted next generation sequencing, a short interspersed nuclear element insertion was identified near a splice acceptor site in an intron of a provocative gene, *FAM161A*. Analysis of mRNA from an affected dog revealed that the SINE causes exon skipping, resulting in a frame shift, leading to a downstream premature termination codon and possibly a truncated protein product. This mutation segregates with the disease in 22 out of 35 cases tested (63%). Of the PRA controls, none are homozygous for the mutation, 15% carry the mutation and 85% are homozygous wildtype. This mutation was also identified in Tibetan Terriers, although our results indicate that PRA is genetically heterogeneous in both Tibetan Spaniels and Tibetan Terriers.

## Introduction

Progressive retinal atrophy (PRA) in animals is the term used for a group of inherited retinal diseases characterised by progressive retinal degeneration resulting in loss of vision. Typically, rod photoreceptor responses are lost first followed by cone photoreceptor responses [1]. Bilateral and symmetrical fundus changes are observed, including tapetal hyper-reflectivity in the early stages followed by vascular attenuation, pigmentary changes and atrophy of the optic nerve head in the later stages of disease [2]. Forms of PRA have been documented in more than 100 dog breeds and while they exhibit similar clinical signs, the aetiology, age of onset and rate of progression vary between and within breeds. While several disease-causing genes have been reported for some forms of PRA [3], many remain undefined. Retinitis Pigmentosa (RP), the human equivalent of PRA, is the collective name for a group of inherited human retinal disorders that lead to progressive loss of vision in approximately 1 in 4000 people [4]–[6]. As in PRA, rod photoreceptor cells are predominantly affected, resulting in clinical symptoms that typically include night blindness and loss of peripheral vision. However, the cones also degenerate; resulting in central vision loss and eventually complete blindness can result. To date, at least 192 genes have been shown to cause a wide spectrum of retinal disease, including RP (RetNet; http://www.sph.uth.tmc.edu/retnet/), although mutations in these genes currently only account for approximately 30% of recessive RP cases. [7].

Canine diseases are valuable natural models for the study of many varied human conditions such as autosomal recessive congenital ichthyosis [8], myotubular myopathy [9] and hereditary retinopathies such as Leber congenital amaurosis (LCA) and achromatopsia [10], [11]. Further to this, canine models for human eye diseases have proved invaluable in gene-therapy studies, most notably the canine models of LCA associated with *RPE65* [12]–[17] and X-linked RP associated with *RPGR* [18].

Most PRA cases in the Tibetan Spaniel (TS) are clinically indistinguishable from other forms of PRA. The mode of inheritance appears from pedigree information to be autosomal recessive and the age of diagnosis is relatively late, typically at approximately 5 years of age [19]. No mutations have previously been associated with PRA in the breed.

Here we report the identification of a short interspersed nuclear element (SINE) insertion in *FAM161A*, a ciliary gene previously associated with RP in humans [20], [21]. The mutation causes exon skipping and a subsequent shift in the reading frame resulting in a premature termination codon. We present evidence that this mutation represents a major susceptibility locus for late onset PRA, referred to hereafter as PRA3, in TS and Tibetan Terriers (TT).

## Results

### Genome–wide association mapping

**Figure 1:**
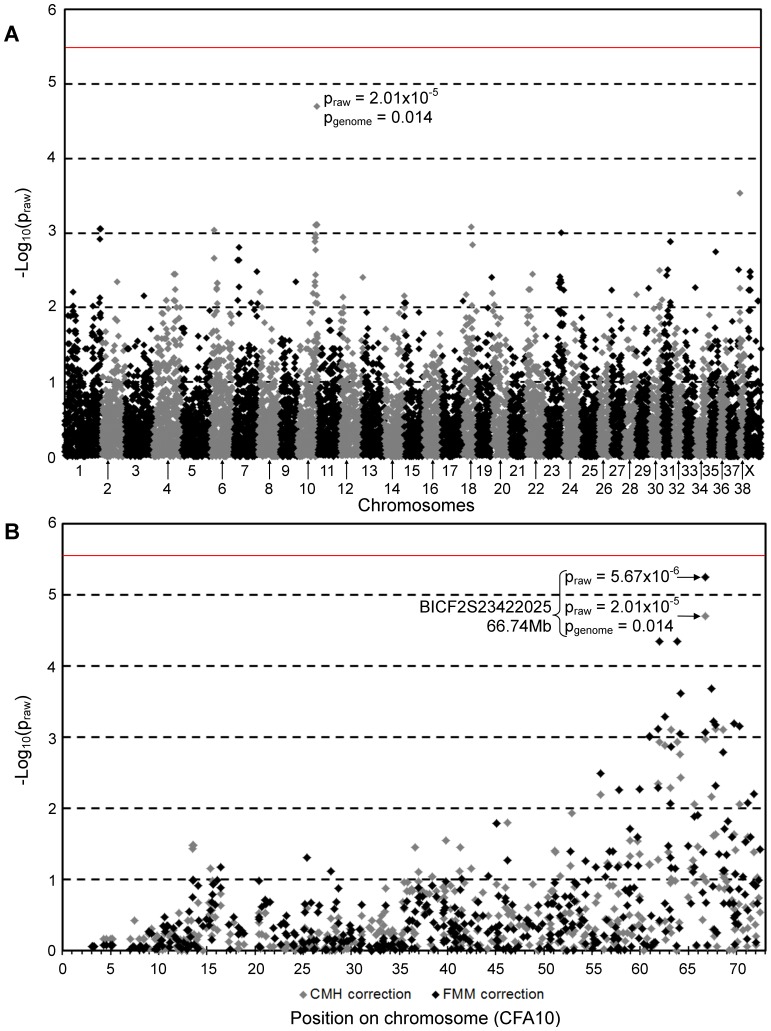
Genome-wide association mapping of PRA in Tibetan Spaniels. -Log_10_ of p-values after correction for population stratification. The red lines indicate the Bonferroni-corrected 5% significance level based on 15,674 SNPs. **A)** The CMH meta-analysis approach shows the strongest signal on CFA10 (p_raw_ = 2.01×10^−5^, P_genome_ = 0.014). **B)** The signal spans a region of 5.37 Mb from 62 to 67.37 Mb on CFA10.

Genome-wide association (GWA) analysis of genotyping data from 32 TS dogs (22 cases and 10 controls – seven over the age of six and three over the age of four when last examined) genotyped with 15,674 SNPs revealed a genome-wide significant association on chromosome 10 (CFA10; p_raw_ = 1.77×10^−7^, p_genome_ = 0.004). Two SNP markers 1.86 Mb apart (BICF2P729624 at 62.0 Mb and BICF2S23250878 at 63.86 Mb) were equally the most associated with PRA. Identity-by-state (IBS) clustering confirmed the presence of population stratification with a high genomic inflation factor, λ = 1.69. The inflation factor was reduced to an acceptable level (λ = 1.06) after correcting for population stratification [22]. While the signal on CFA10 (p_raw_ = 2.01×10^−5^, Figure 1A) dropped below the level of Bonferroni-corrected significance, the permutation-corrected signal remained statistically associated (p_genome_ = 0.014). Alternative analysis of the data using Fast Mixed Model (FMM) [23] revealed similar results (Figure S1). While only the most associated SNP (BICF2S23422025 at 66.74 Mb: p_genome_ = 0.014) was statistically associated, the signal on CFA10 extended from approximately 62 to 67 Mb (Figure 1B), defined using SNPs with p_raw_<10^−3^.

### Haplotype and homozygosity analysis

**Figure 2:**
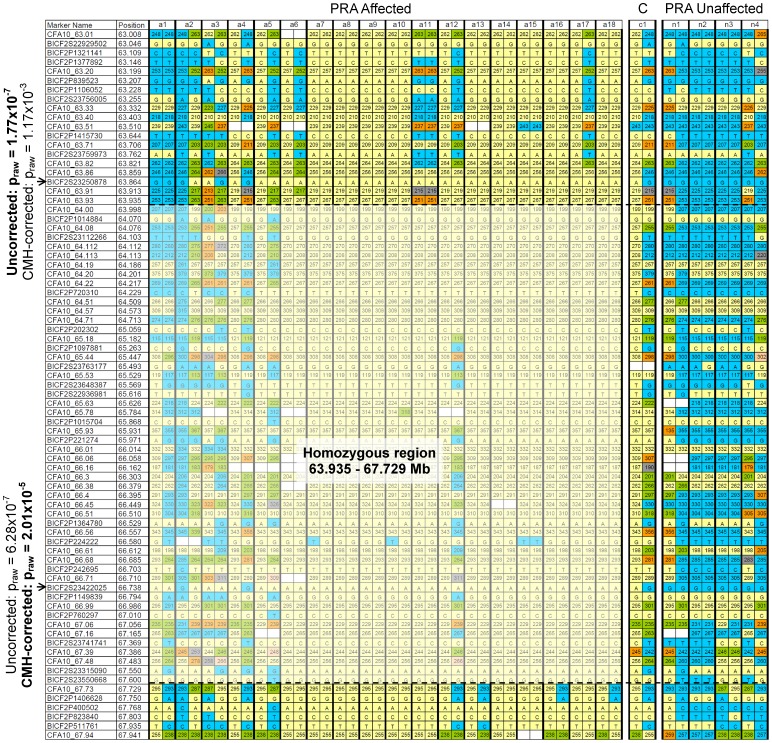
Critical region definition using homozygosity analysis. Microsatellite and SNP marker genotypes for 18 PRA cases and five PRA controls (including one obligate carrier) over the region identified during the GWA study. It is not possible to define a region for which all of the cases are homozygous, however, it is possible to define a broad region for which most of the cases (12/18) are homozygous, from 63.935 to 67.729 Mb. The most associated SNP markers are indicated with arrows (→).

A haplotype homozygous in all cases, but not in controls, could not be easily identified through homozygosity analysis (data not shown). The most highly-associated SNP, BICF2S23422025 (p_genome_ = 0.014) is homozygous (A/A) in most of the cases (19/22), but also in 3/10 controls. Due to the low resolution of the SNP20 BeadChip, only 15,674 SNPs were informative in the TS cohort, resulting in 1 SNP approximately every 159 kb. This made it difficult to identify homozygous haplotypes from the SNP data alone and additional microsatellite markers from the region were therefore genotyped to provide additional haplotype information. Due to limited sample availability only eighteen of the original 22 cases and five of the original ten controls (one of which is an obligate carrier) used in the GWA study were used in the microsatellite marker genotyping study (Figure 2). It was not possible to define a haplotype that was homozygous in all cases, although a broad critical region of 3.794 Mb, from 63.935 Mb to 67.729 Mb on CFA10 was identified. This region is almost completely homozygous in the majority of cases (12/18) and none of the controls, is heterozygous in the obligate carrier, and contains 31 genes, 29 of which have human orthologues.

At the time this work was undertaken, none of the genes in the region could be identified as strong functional candidates. However RP28 had been mapped to a locus in humans (2p14-15) [24], [25], and part of the genomic region in dogs syntenic to the RP28 locus overlaps with the PRA critical region identified in this study. We opted to investigate this defined region using targeted resequencing.

### Next generation sequencing

**Figure 3:**
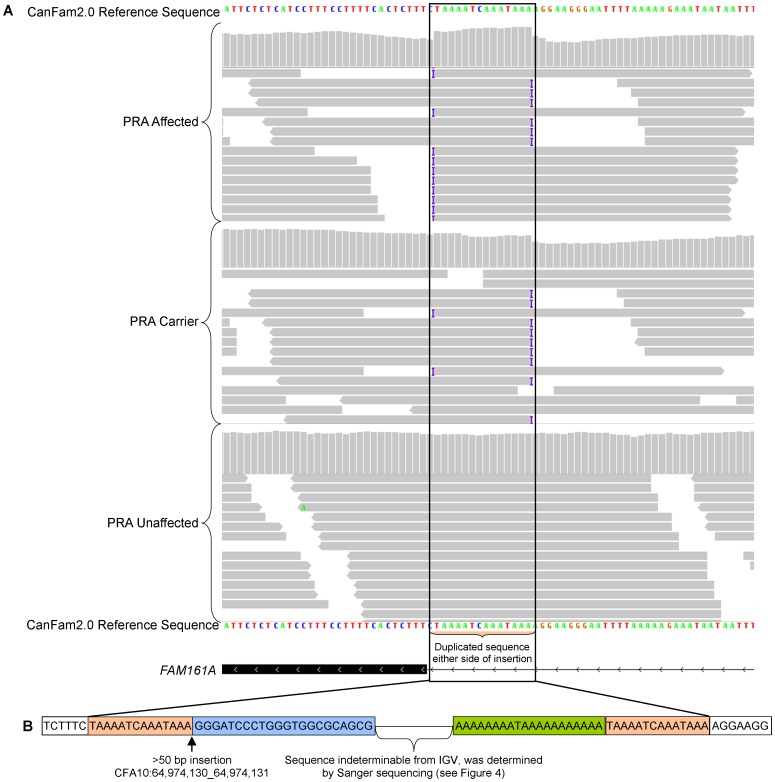
IGV display of the SINE insertion in *FAM161A*. A) Each of the three samples (PRA-affected, obligate carrier and control) viewed in IGV are represented by two panels. The sudden change in read depth (vertical bars in upper panels) in the affected dog is characteristic of a duplication, caused by the repeat motif flanking the insertion. The sudden termination of reads (horizontal bars in lower panels) and the insertion symbol (**I**) either side of the duplicated sequence is also characteristic of an insertion flanked by the duplicated sequence. The inserted sequence is present in all reads in the PRA-affected dog, approximately half the reads in the obligate carrier and none of the reads in the PRA-unaffected (control) dog. B) Inserted sequence (blue and green) as determined from NGS data, flanked by 14 bp repeats (orange).

To identify potential disease-causing mutations we resequenced the 3.8 Mb critical region in 10 dogs (four PRA affected, two obligate carrier and four unaffected dogs). We identified 19,111 SNPs and 3,740 insertion-deletions (indels) when compared with the CanFam2 reference sequence. Of these, 194 SNPs and 81 indels segregated with the phenotype, but none of these variants were predicted to alter a protein product. Visual analysis of sequence data in the Integrated Genome Viewer (IGV) revealed 16 additional variants (indels>10 bp were not reliably identified using our analysis pipeline), of which seven segregated with the phenotype, but only one was in or near an exon. This latter variant was an insertion flanked by a 14 bp repeat motif (Figure 3), visualised in IGV as a change in the read depth. The length of the inserted sequence is longer than the length of the NGS reads (>50 bp) and the precise sequence of the insertion could therefore be only partly determined (Figure 3B). Only this variant, which was predicted to be located near a splice acceptor site of the *FAM161A* gene (CFA10: 64,974,130), was predicted to alter a protein product, by interfering with exon splicing.

**Figure 4:**
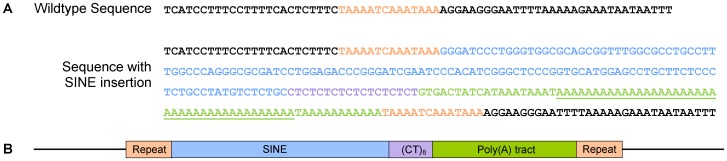
Sequence of the SINE insertion. A) The sequence and B) graphical representation of the SINE insertion. The precise number of nucleotides that comprise the underlined portion of the poly(A) tract remains unclear, but is approximately 35–50.

The full sequence of the insertion was determined by Sanger sequencing using primers flanking exon 5 of *FAM161A*, including the insertion site in genomic DNA (gDNA) from 80 TS dogs (29 affected with PRA, 10 obligate carriers and 41 unaffected) (Figure 4). Using agarose gel electrophoresis, a single band of the expected size (720 bp) was visible for 40 unaffected samples, while a band approximately 230 bp larger (∼950 bp) was visible for 17 of the PRA affected samples and none of the unaffected samples (data not shown). Sequencing of the ∼230 bp insertion revealed that it contains a 132 bp SINE–a retrotransposon that is distributed widely throughout the canine genome [26]. Characteristically, the SINE is followed at the 3′ end by a dinucleotide repeat (CT)_8_, and a poly(A) tract (interrupted by the occasional T) at least 45 bp in length. The nucleotides at the 3′-end of the poly(A) tract are duplicated at the 5′-end of the SINE (Figure 4). The precise number of adenine nucleotides that comprise a portion of the poly(A) tract (underlined in Figure 4A) could not be determined accurately due to difficulties amplifying homopolymers with synthetic taq polymerases, specifically polymerase slippage along the poly(A) tract. However, based on the sequence traces, there appear to be 35–50 adenine nucleotides. This is consistent with the insertion size of ∼230 bp as estimated from the bands observed on the agarose gels. There is limited evidence that the poly(A) tract length is variable between the cases, but this does not affect the severity of the phenotype, although further investigation is required (data not shown).

### Transcript evaluation and comparison

**Figure 5:**
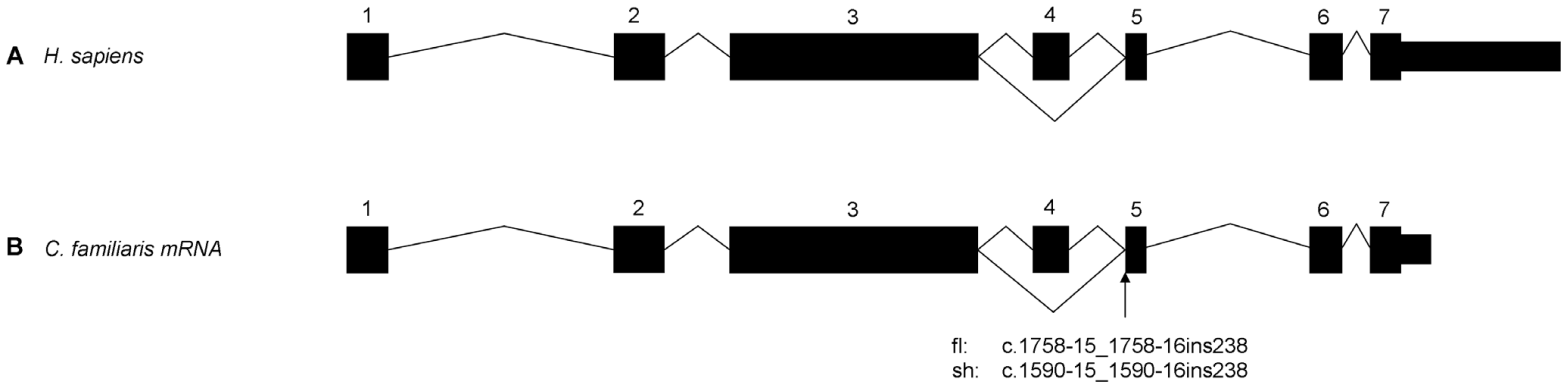
Graphical comparison of the intron-exon boundaries of FAM161A. A) Human (Homo sapiens) *FAM161A*. B) Canine *FAM161A* exons confirmed by sequencing the retinal mRNA transcript. The location of the splice site insertion is indicated.

In humans there are two main *FAM161A* isoforms, full-length (*FAM161A*_fl_) and short (*FAM161A*_sh_), formed by alternative splicing of exon 4 (Figure 5A) [20], [21]. The coding sequence of the *FAM161A* retinal transcripts, from both the main isoforms (*FAM161A*_fl_ and *FAM161a*_sh_), was successfully sequenced in a healthy dog, excluding the first 46 nucleotides of the coding sequence (Figure S2). Sequencing revealed that both isoforms are transcribed in the canine retina. In addition, intron-exon boundaries are identical to those of the human and mouse, which is in conflict with the boundaries predicted by Ensembl (CanFam2.0) genebuild for the canine gene (Figure 5B and Figure S2). Sequencing of the full 5′ and 3′ UTRs and the beginning of exon 1 was unsuccessful, likely due to high GC content. Sequencing revealed that canine *FAM161A*_fl_ contains 716 amino acids (Genbank accession no. KF177335) and *FAM161A*_sh_ contains 660 amino acids (Genbank accession no. KF177336), with predicted molecular weights of 83 kDA and 76 kDa respectively. The SINE insertion occurs near the acceptor splice site of intron 4; i.e. near the boundary of intron 4 and exon 5 (*FAM161A*_fl_: c.1758-15_1758-16ins238; *FAM161A*_sh_: c.1590-15_1590-16ins238) (Figure 5B).

**Figure 6:**
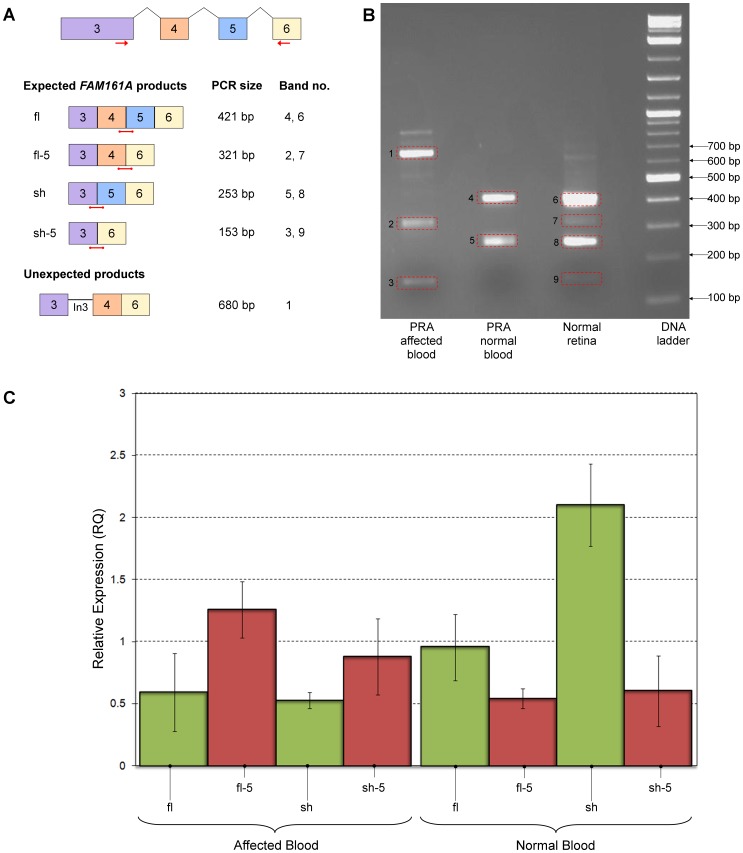
Comparison of *FAM161A* mRNA isoforms. PCR, electrophoresis and qRT-PCR to compare the *FAM161A* isoforms in blood from affected and unaffected dogs. A) Primers in exons 3 and 6 (red arrows) were used to amplify *FAM161A* isoforms created by alternative splicing of exons 4 and 5, resulting in four possible amplicons expected (fl, fl-5, sh, sh-5; sizes indicated). Red bars represent isoform-specific qPCR probes. B) Agarose gel electrophoresis of PCR amplicons. C) Relative expression of each target (wildtype targets in green and mutant targets in red), determined by qPCR. Error bars represent the standard deviation.

The location of the SINE insertion near the splice acceptor site of exon 5 suggests that exon splicing may be affected, possibly resulting in the skipping of exon 5. To assess this hypothesis, mRNA transcripts were compared between a TS dog homozygous for the SINE insertion and two dogs of unknown breed homozygous for the wildtype allele. In the absence of suitable retinal tissue, RNA was purified from the blood of the affected and one of the unaffected dogs, while retinal tissue was available from the second unaffected dog. Primers in exons 3 and 6 were used to amplify across exons 4 and 5. A number of products were produced for all three samples, each of which was individually sequenced (Figure 6).

Bands 4 and 6 comprised the 421 bp amplicon (*FAM161A*_fl_), and bands 5 and 8 comprised the 253 bp amplicon (*FAM161A*_sh_). Both of these isoforms were detectable in unaffected blood and retina, but not blood from the affected dog.

Bands 2 and 7 comprised the 321 bp amplicon (*FAM161A*_fl-5_) and bands 3 and 9 comprised the 153 bp amplicon (*FAM161A*_sh-5)_. Both of these amplicons were detectable in blood from the affected dog. Interestingly these bands were also detectable in unaffected retina, albeit at lower levels than *FAM161A*_fl_ and *FAM161A*_sh_, but not blood from the unaffected dog. These observations suggest that in normal dogs natural splicing of the *FAM161A* gene results in low levels of transcripts lacking exon 5, in addition to the predominant wildtype transcripts. The levels of the mutant transcripts in blood from an unaffected individual are likely too low to be detected by these methods.

Band 1 comprised an amplicon containing *FAM161A*_fl-5_ and intron 3, which could be a result of gDNA contamination or incomplete or inaccurate exon splicing. While not visible in Figure 6B, a band comprising FAM161A_fl_ and intron 3 was also detected in unaffected blood during similar assays. This inconsistent observation is likely due to the target being present at exceedingly low levels in the blood.

Quantitative reverse transcription PCR (qRT-PCR) was also used to compare the levels of mutant (fl-5 and sh-5) and wildtype (fl and sh) transcripts between the “affected” and “unaffected” blood samples (Figure 6C). The mutant transcripts are the predominant species in the affected sample while the wildtype transcripts are predominant in the unaffected sample.

These results indicate that the difference between *FAM161A* in PRA3 affected and unaffected blood is the absence and presence respectively of exon 5, supporting the exon-skipping hypothesis.

### Mutation screening

All 32 TS dogs (22 cases and 10 controls) that participated in the GWA study were screened for the SINE insertion (*FAM161A*_c.1758-15_1758-16ins238_) to compare it with two of the most highly associated SNP markers, BICF2S23422025 and BICF2S23250878. *FAM161A*_c.1758-15_1758-16ins238_ showed significant allelic association with PRA (p_raw_ = 5.03×10^−7^). The SNP markers also showed significant allelic association, although BICF2S23422025 was less associated (p_raw_ = 6.28×10^−7^). However, BICF2S23250878 was more highly associated (p_raw_ = 1.77×10^−7^) than *FAM161A*_c.1758-15_1758-16ins238_, but this can be attributed to two PRA cases that are heterozygous for the SNP (i.e. carry the minor allele) but are homozygous for the wildtype *FAM161A* allele. The small sample size used along with possible genetic heterogeneity with the sample set is likely to have contributed to this unexpected observation. As we have no reason to believe that this form of PRA has anything other than a recessive mode of inheritance, these data do not warrant the elimination of the *FAM161A* variant from further investigation. Fifteen out of 22 PRA cases and none of the controls were homozygous for *FAM161A*_c.1758-15_1758-16ins238_. Analysis of the segregation of *FAM161A*_c.1758-15_1758-16ins238_ with PRA within a family of 48 dogs, including seven cases (Figure S3), indicates that the form of PRA associated with this variant is recessive and fully penetrant. The form of PRA that is associated with *FAM161A*_c.1758-15_1758-16ins238_ is known hereafter as PRA3.

To confirm that the variant is not a commonly occurring polymorphism in this breed, we screened 215 additional TS dogs, resulting in a total of 247 TS tested for *FAM161A*_c.1758-15_1758-16ins238_ (Table 1). Of the 35 PRA cases used in the study 22 (62.9%) were homozygous for *FAM161A*_c.1758-15_1758-16ins238_ (*FAM161A* ^−/−^) and all 116 dogs known to be clinically free of PRA at their last eye examination, including 16 obligate carriers of PRA, were either carriers of the mutant allele (14.7%; *FAM161A* ^+/−^) or homozygous for the wild type allele (85.3%; *FAM161A* ^+/+^). PRA3 therefore accounts for the majority of cases of PRA in our TS cohort.

**Table 1:** PRA3 genotypes and PRA clinical status for 247 TS.

	PRA Clinical Status				
Genotype^i^	PRA Case	PRA Carrier	Clear	Unknown UK	Total
**FAM161A^−/−^**	22 (62.9%)	0 (0%)	0 (0%)	2 (2.1%)	24
**FAM161A^+/−^**	2 (5.7%)	9 (56.3%)	8 (8.0%)	22 (22.9%)	41
**FAM161A^+/+^**	11 (31.4%)	7 (43.8%)	92 (92.0%)	72 (75.0%)	182
**Total**	35	16	100	96	247

i The wildtype allele is represented by “+” and the mutant allele by “−”.

To determine whether *FAM161A*_c.1758-15_1758-16ins238_ is associated with PRA in related breeds we screened a further 99 dogs from two closely related breeds most likely to share polymorphisms with the TS breed. These were 23 Lhasa Apsos (LA) and 76 TT (data not shown), including nine LA and 12 TT affected with PRA. All 23 LA dogs, including nine PRA cases, were homozygous for the wild-type allele (*FAM161A* ^+/+^). PRA3 is therefore absent from this LA cohort, but as the number of dogs tested was small, it is not possible to exclude the possibility that PRA3 does exist as a rare form of PRA within the LA breed. Of the 12 TT with PRA, four were homozygous for *FAM161A*_c.1758-15_1758-16ins238_ (*FAM161A* ^−/−^), while the remaining eight PRA cases were either heterozygous (*FAM161A* ^+/−^; n = 1) or homozygous wildtype (n = 7). In addition, all TT known to be free of PRA (n = 10) were homozygous for the wildtype allele (79.7%). PRA3 is therefore present in the TT breed.

AHT Genetic Services have tested 567 TS and 290 TT for PRA3 over the course of 10 months and six months, respectively and these data indicate an allele frequency of 0.052 in TS and 0.0052 in TT worldwide (Nigel Holmes, personal communication).

## Discussion

Using a GWA mapping and homozygosity analysis approach, a novel 3.8 Mb locus on chromosome 10 that is associated with PRA in the TS was identified. This region overlapped with part of the region syntenic to the human RP28 locus [24]. The entire critical region was sequenced and a single provocative variant was identified in the *FAM161A* gene. *FAM161A* was subsequently identified as a strong positional candidate causal locus due to the identification of *FAM161A* mutations in humans with RP and the localisation of FAM161A to the photoreceptors of the retina [20], [21].

The average age-at-diagnosis of all PRA cases in our cohort (including non-PRA3 and excluding obvious outliers at 8.3, 10.0, 10.2 and 11.3 years) is 4.8 years. Therefore, suitable controls should have been at least 6 years old. At the time the GWA mapping was undertaken we had very few robust control samples available i.e. with clear eye examinations. As result the best control cohort we could collect was made up of seven dogs over the age of six years, which we supplemented with three dogs over the age of four years. It is possible that any of these controls could be incorrectly diagnosed as clear and may in fact develop PRA at a later date, thereby reducing the power of the GWA study. Nevertheless, whilst we acknowledge that older controls should have been used, we decided to proceed with the available cohort. As it turned out, none of 10 controls used were homozygous for the mutation subsequently identified.

*FAM161A* mRNA is expressed in the normal canine retina, the intron-exon boundaries are identical to the human and mouse boundaries and it is alternatively spliced to produce two isoforms, one containing and one lacking exon 4 (*FAM161A*_fl_ and *FAM161A*_sh_ respectively). Sanger sequencing and qPCR results indicate that *FAM161A* mRNA transcripts in healthy retinal tissue and blood from dogs not affected with PRA comprise predominantly the wildtype *FAM161A* isoforms (*FAM161A*_fl_ and *FAM161A*_sh_; Figure 6). Conversely, *FAM161A* mRNA transcripts in blood from a dog affected with PRA3 (i.e. homozygous for *FAM161A*_c.1758-15_1758-16ins238_) comprise predominantly the aberrant *FAM161A* isoforms lacking exon 5 (*FAM161A*_fl-5_ and *FAM161A*_sh-5_). This supports the hypothesis that the SINE insertion results in skipping of exon 5 during pre-mRNA splicing in blood. While it is likely that *FAM161A*_c.1758-15_1758-16ins238_ has the same effect of aberrant splicing in other tissues, the possibly that tissue-specific splicing negates this effect in the retina cannot be excluded. Further investigation using retinal tissue from a dog with PRA3 would be necessary to substantiate the hypothesis of alternative splicing. Interestingly, aberrant *FAM161A* isoforms (*FAM161A*_fl-5_ and *FAM161A*_sh-5_) were also present in retinal tissue from a dog not affected with PRA and homozygous for the wildtype allele, albeit at much lower levels than the wildtype isoforms. These are most likely a result of naturally-occurring alternative splicing, which is a common occurrence. At least 74% of human multi-exon genes are alternatively spliced [27] and up to 30% of alternative transcripts contain premature termination codons [28]. These are usually targets of nonsense-mediated decay (NMD), although Lewis at al observed that 4.3% of RefSeq mRNAs (i.e. experimentally identified mRNAs that have not been degraded) are truncated by >50 amino acids [28]. While these aberrant proteins may well be expressed in healthy retinal tissue, it is clear from data presented here that they are a minor product compared with the normal, functional protein.

**Figure 7:**
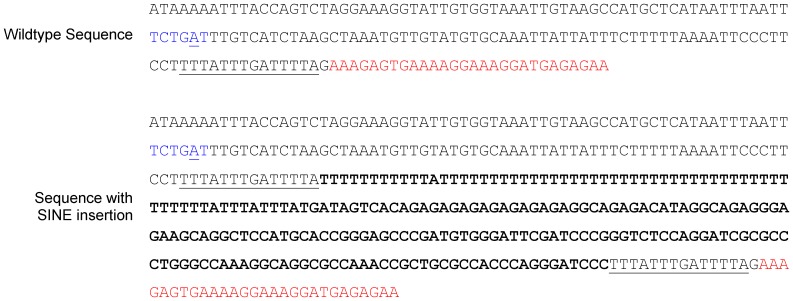
*FAM161A*_c.1758-15_1758-16ins238_ effect on pre-mRNA splicing. Wildtype and *FAM161A* ^−/−^ sequence, with exon 5 (red), intron 4 (grey) and SINE insertion c.1758-15_1758-16ins238 (black) indicated. In the wildtype sequence, the putative BPS (blue, with the critical A underlined) is 76 bp upstream of the intron-exon boundary, while in the affected sequence it is >300 bp upstream of the boundary.

The pre-mRNA splicing mechanism requires at least three consensus intronic sequences for optimal function. One of these is the 3′ consensus sequence 6PyNCAG (where Py is a pyrimidine base, N is any base) of the acceptor site and another, the branch point sequence (BPS), is the site of lariat formation [29]. In eukaryotes the latter is typically, but not always, 20 to 50 nucleotides upstream of the splice junction. The consensus sequence of the BPS, to which the U2 component of the spliceosome binds, is also variable (PyXPyTPuAPy) (Pu is a purine base), although the adenine base is of primary importance for lariat formation [30], [31]. There is no sequence within 50 nucleotides of the *FAM161A* intron 4-exon 5 splice site that corresponds to the BPS consensus sequence. However, a putative BPS located 76 nucleotides from the splice site, does correspond to the consensus sequence (Figure 7). While the SINE insertion does not affect the AG sequence of the acceptor site it will push the BPS beyond its optimal position relative to the acceptor site, which is likely to be the cause of aberrant splicing of exon 5 in affected dogs.

Similar instances of aberrant splicing due to SINE insertions that are associated with canine traits have been reported: An insertion 35 bp upstream of an accepter site of the *HCRTR2* (hypocretin (orexin) receptor 2) gene has been associated with canine narcolepsy in the Doberman breed [32], and an insertion nine bp upstream of an acceptor site of the *SILV* (a.k.a *PMEL;* premelanosome protein) gene has been associated with the merle pigmentation pattern in multiple dog breeds [33].

The broad range of ages-at-diagnosis observed in PRA-affected dogs homozygous for *FAM161A*_c.1758-15_1758-16ins238_ suggests that there may be a great deal of variation in the age-of-onset or severity of PRA3. Variable poly(A) tract length has been shown to affect disease severity [33]–[35], but the limited variation observed within our study suggests that it is unlikely that the poly(A) tract length variation has much effect on the PRA phenotype or severity. However, the number of samples for which age-at-diagnosis and poly(A) tract length is known is small, and a larger sample set is required to support this hypothesis. The observed variation is probably largely due to a cohort that has not been regularly examined and as a result the diagnoses are made at varying stages of disease progression. Some of the variation could be “real”, but this can only be confirmed by studying a colony of dogs bred specifically to study this disease, and which are closely monitored.

In order to further test the validity of the insertion variant, *FAM161A*_c.1758-15_1758-16ins238_, we screened 247 TS for the variant (Table 1). We found that 62.9% of the PRA cases, 56.3% of the obligate PRA carriers and 100% of clinically unaffected dogs (which could be clear of the variant or carry a single copy) have *FAM161A* genotypes that are concordant with their clinical status. There are two groups of dogs with genotypes discordant with their phenotypes. The first comprises two dogs that are homozygous for the variant and have not been diagnosed with PRA. Clinical information pertaining to one of these dogs was unavailable, although it is known to have had at least one PRA-affected sibling. The other dog had not been examined by an ophthalmologist but its owner reported no significant loss of sight by the time it died. However, it had lost one eye in an accident and developed a cataract in the other eye around nine years of age, which could have been secondary to PRA. The observation that 91.7% (22/24) of dogs homozygous for *FAM161A*_c.1758-15_1758-16ins238_ (i.e. *FAM161A* ^−/−^) have developed PRA suggests the variant is fully penetrant, or nearly so. The inheritance observed in a family of 48 dogs (seven cases) is supportive of a recessive mode (Figure S3B). The second group of discordant dogs comprises 13 PRA-affected dogs that are not homozygous for *FAM161A*_c.1758-15_1758-16ins238_ and seven obligate carriers do not carry *FAM161A*_c.1758-15_1758-16ins238_. Not all of these dogs are in families distinct from those in which PRA3 segregates (Figure S3A). It is possible that the variant has a dominant mode of inheritance with incomplete penetrance, or complex trait or compound heterozygote effects. However, as heterogeneity of PRA has been seen in other breeds [36], [37] it is more likely that additional loci are responsible for the discordant cases.

Anecdotal evidence of their shared origins in Tibetan monasteries suggests that TT and LA are the most closely related breeds to the TS, and as a result these are the breeds most likely to share the PRA3 variant. Screening of 76 TT and 23 LA dogs, including 12 and nine PRA cases respectively, revealed that the variant is present in TT, but is absent from the LA screened. *FAM161A* genotypes of 33.3% of the TT PRA cases and 100% of clinically unaffected dogs were concordant with their clinical status (data not shown). Interestingly, two of the eight PRA-affected TT that were not homozygous for *FAM161A*_c.1758-15_1758-16ins238_ (i.e. *FAM161A* ^+/−^ or *FAM161A* ^+/+^), were in fact homozygous for the mutation associated with RCD4 [36]. It is therefore likely that PRA in the remaining six cases in the breed is caused by a third unknown mutation.

*FAM161A* encodes the family with sequence similarity 161, member A protein and was recognised as an appealing candidate gene due to its involvement in RP in humans [20], [21]. The gene occurs in two main isoforms that differ by the presence or absence of exon 4 (*FAM161A*_fl_ and *FAM161A*_sh_ respectively) [20]. Both isoforms are expressed in multiple tissues including the retina and testes, and at lower levels in the heart, liver, kidney, brain, muscle, lung and thyroid gland [21]. Specifically, the protein has been localised to the connecting cilium and basal body in the inner segment of rod and cone photoreceptor cells, and to the basal body and centrosome of ciliated cells of different origins [38], [39]. *FAM161A* has been shown to interact with the CRX (Cone-rod homeobox-containing) transcription factor [21] and Lebercilin [38], both of which have also been implicated in retinal degeneration in humans [40], [41]. Only a single evolutionary conserved domain (UPF0564) has been identified, which is vital for binding to and stabilising microtubules [20], [39]. This region is also required for homotypic FAM161A interactions, as well as heterotypic interactions with paralog FAM161B (family with sequence similarity 161, member B) [39]. FAM161B interacts with TACC3 (transforming, acidic coiled-coil containing protein 3), which in turn is involved in centrosome-dependent microtubule assembly, kinetochore attachment, chromosome alignment and mitotic exit [42]. FAM161A could therefore be involved in maintenance of the microtubule axoneme along the connecting cilium or protein transport between the inner segment and outer segment of the photoreceptors [38], [39].

*FAM161A*_c.1758-15_1758-16ins238_ affects splicing of exon 5 of the gene, which shifts the reading frame resulting in 13 aberrant amino acids and a truncated protein. The loss of 166 amino acids includes approximately 44 amino acids of the UPF0564 conserved domain. However, Bandah-Rozenfeld et al reported that the N-terminus of the UPF0564 domain is sufficient for homotypic and heterotypic interaction with FAM161B [20]. The truncated protein product is therefore expected to be functional in this regard. As the discovery of *FAM161A* involvement in retinal disease was relatively recent, little is known about the protein's structure and function of the protein in visual pathways. Further investigations are required to elucidate the precise pathways in which FAM161A is involved, which may lead to the identification of novel functional domains in the C-terminus of the protein. To this end, the canine model described here could be particularly useful, as no other animal models have been reported.

The presence of *FAM161A* mutant mRNA transcripts in the blood of an affected dog implies that the truncated transcript is not subjected to nonsense-mediated decay. A truncated protein may therefore be expressed, although this would need to be confirmed by comparing FAM161A protein levels in *FAM161A* ^−/−^ dogs with protein levels in *FAM161A* ^+/+^ dogs. If this is the case, the truncated protein product must be sufficient to cause retinal degeneration.

PRA3, caused by the variant described here, has an average age at diagnosis of 4.89 years and this is indicative of a late age of onset (data not shown). It is important to note, however, that this is not necessarily an accurate estimation of the age of onset of the disease. Many breeders may avoid having their dogs screened until they are pressured by breeders involved in research projects such as this, or until they notice obvious signs that their dog has visual problems, by which point the disease is often advanced. It is therefore possible, if not highly likely, that the age of onset of PRA3 is much earlier than the estimated 4.89 years. Nevertheless, these results are consistent with observations in human patients in which the age of onset was in the 2^nd^ or 3^rd^ decade [21]. Given that FAM161A is expressed in multiple tissues, it would be interesting to determine whether a more severe change to the protein, such as a knock-out, would result in a more severe retinal or even systemic phenotype. The discordant TS PRA cases i.e. *FAM161A* ^+/+^ and *FAM161A* ^+/−^ tended to develop PRA at a later age, with an average age at diagnosis of 7.01 years (data not shown), which is consistent with the segregation of a second form of PRA in the TS breed.

PRA in the TS has not previously been associated with any genetic variants. Using a GWA mapping approach, a novel candidate variant, *FAM161A*_c.1758-15_1758-16ins238_, was identified that is likely to represent a major causal mutation for PRA in the TS. While this mutation does not account for all cases of PRA in this study, suggesting that there are additional loci causing PRA in this breed, it does appear to be highly penetrant and a major cause of PRA in this breed. While PRA3 is also present in TT, as they are closely related and the mutation has not been found in any other breeds, the mutation appears to be confined to these two breeds.

## Materials and Methods

### Sample collection and processing

The diagnosis of individual dogs was determined by veterinary ophthalmologists independently, or through the BVA/KC/ISDS (British Veterinary Association/Kennel Club/International Sheep Dog Society) Eye Scheme in the UK. Cases were defined as dogs diagnosed as affected with PRA i.e. displaying ophthalmascopic signs of PRA including tapetal hyperreflectivity and vascular attenuation. Controls were those free of inherited eye disease of any kind, and at least 4 years old at the time of examination for the GWA analysis or any age for subsequent investigations.

Blood samples were collected into EDTA tubes and genomic DNA was extracted from whole blood using a Nucleon Genomic DNA Extraction Kit (Tepnel Life Sciences), according to the manufacturer's instructions. For samples collected as buccal mouth swabs, DNA was extracted using a QIAamp DNA Blood Midi Kit (Qiagen). A canine retinal tissue sample from a dog of unknown breed and free of PRA was taken post mortem, with the owner's consent, and preserved in RNAlater (Life Technologies). RNA was extracted using an RNeasy Protect Mini Kit (Qiagen) according to the manufacturer's instructions.

Blood samples from two dogs (a TS with PRA and homozygous for the SINE insertion, and from a dog of unknown breed but free of PRA and homozygous for the wildtype allele) were collected into EDTA tubes. RNA was extracted using the PerfectPure RNA Blood Kit (5 Prime) or the QIAamp RNA Blood Mini Kit (Qiagen) according to the manufacturers' instructions.

### SNP genotyping and genome-wide association mapping

Canine SNP20 BeadChips (Illumina) were used to obtain genotype calls for 22,362 single nucleotide polymorphisms (SNPs) using DNA from 22 TS PRA cases and 10 TS controls (seven over the age of 6 years and three over the age of 4 years) and GWA analysis was conducted using the software package PLINK [22]. After removing SNPs with a minor allele frequency <5% and missing genotype calls >10% from the analysis, a final data set of 15,674 markers remained. Sample call rate was >99.7% for all samples. IBS clustering and Cochran-Mantel-Haenszel meta-analysis with PLINK were used to examine and adjust for population stratification [22]. A mixed model analysis using Fast Mixed Model [23] was also undertaken to correct for population stratification. As a correction for multiple testing, we repeated the GWA analyses using the Max(T) permutation procedure in PLINK (100,000 permutations). P-values generated before multiple testing correction are denoted by p_raw_, while those generated after are denoted by p_genome_.

### Microsatellite marker genotyping

Microsatellite markers within the associated region were genotyped in 18 cases and 5 controls used in the GWA investigation. Primers flanking each marker were designed using Primer3 [43] and PCR was used to amplify the target DNA using 12 uL reactions (Table S1). The products were separated by size on a 3130xl Genetic Analyzer (Applied Biosystems) and the data analysed and alleles assigned to each sample with the GeneMapper software package (Applied Biosystems). Visual inspection of SNP and microsatellite marker genotypes and haplotypes across the region was performed to define a homozygous critical region.

### Next generation sequencing

Genomic DNA (3 μg) from 10 TS dogs (four PRA-affected, two obligate carrier and four PRA-clear) was used to prepare libraries for sequencing, using the SureSelectXT Custom MP4 Kit (Agilent Technologies). This kit contained a custom capture library of 40,473 biotinylated RNA baits 120 bp in length and designed based on the CanFam2.0 reference sequence (CFA10:63–65 Mb) using the Agilent Technologies eArray tool [44]. Baits were designed to give 2X coverage and to exclude repeat-masked regions, resulting in coverage of 54.5% (2.72/5 Mb) of the targeted region. Target enrichment was performed according to the manufacturer's instructions. Initial shearing of genomic DNA using a Covaris S220 and quality assessment of the final library using a 2100 Bioanalyser was undertaken by The Eastern Sequence and Informatics Hub (EASIH, University of Cambridge). The quantity of the captured library was assessed by quantitative PCR using the KAPA Library Quantification Kit for the Illumina Genome Analyzer Platform (KAPA Biosystems), according to the manufacturer's instructions.

Paired-end sequencing resulting in 51 bp reads was conducted in a single lane on an Illumina HiSeq 2000, by the High Throughput Group (HTG) at the Welcome Trust Centre for Human Genetics, University of Oxford, UK. Sequence reads were aligned with the CanFam2.0 canine reference sequence using BWA [45], variant (indel and SNP) calls were made using GATK [46] and aligned reads were visualised using the Integrative Genomics Viewer (IGV) [47]. More than 193 million reads were generated across all 10 samples (representing a 9.9 Gb dataset), of which 72% were mapped to the targeted region on CFA10. The average read depth across the targeted region for all samples ranged from 102X to 174X, and approximately 65% of the region covered by baits was sequenced with at least 30X coverage. Variants considered as candidates for further investigation were those that occurred in splice sites or could affect splicing, or resulted in non-synonymous changes to a protein, and were homozygous in PRA cases, heterozygous in obligate carriers and homozygous for the wildtype allele in controls.

### Primers for sequencing and variant genotyping

The exon-intron boundaries of canine *FAM161A* were defined by producing ClustalW [48] alignments using the Ensembl predicted canine transcripts (ENSCAFG00000003079) and available known mouse (NSMUSG00000049811) and human (ENSG00000170264) Ensembl transcripts. Primer3 [43] was used to design all primers (Table S2), fluorescent and non-fluorescent (Integrated DNA Technologies). These included primers in the exons for the amplification and sequencing of cDNA; in the introns flanking exon five for the amplification and sequencing in genomic DNA; and fluorescent allele-specific primers to detect the presence or absence of the insertion. Amplification products generated using fluorescent primers were used for subsequent fragment length polymorphism detection using an ABI 3130xl DNA Analyzer and GeneMapper Software (Applied Biosystems).

### DNA and RNA sequencing

*FAM161A* complimentary DNA (cDNA) was generated using SuperScriptII Reverse Transcriptase (Invitrogen) or SuperScript VILO Mastermix (Life Technologies) according to the manufacturers' instructions. The region containing the SINE insertion was amplified from gDNA and the entire gene from cDNA (Table S2). PCR products were purified using Multiscreen HTS-PCR filter plates (Millipore). Amplification products were sequenced on an ABI 3130xl DNA Analyzer using BigDye Terminator v3.1 (Applied Biosystems) and sequence traces were assembled, analysed and compared using the Staden Package [49].

### qRT-PCR

Quantitative PCR assays were carried out on an Illumina Eco machine in 20 μL reactions (Table S3). In order to create template/target DNA for standard curve generation for the unknown assays the four possible targets (fl, fl-5, sh and sh-5) were amplified from “affected” (homozygous for the SINE insertion) or “unaffected” (homozygous for the wildtype allele) blood cDNA using PCR (as described above using HotStarTaq Plus DNA Polymerase). PCR products were ligated into pCR2.1 plasmid vector and transformed into OneShot TOP10 Chemically Competent *E.coli*, both part of the TA Cloning Kit (Invitrogen), according to the manufacturer's instructions. Target plasmids were identified using PCR and isolated using the PureLink Quick Plasmid Miniprep Kit (Invitrogen). A mixture of affected and unaffected blood cDNA was used as the template for standard curve generation of the control (*ACTB* and *TBP*) assays. Reaction efficiencies were calculated from a standard curve created using a seven point 2X serial dilution of blood cDNA or plasmids containing the target. Reaction efficiencies were estimated and standard curve r2 values were all >0.99 (Table S3).

### AFLP and variant genotyping

To further investigate the variant (*FAM161A*_c.1758-15_1758-16ins238_), the 32 TS (22 cases and 10 controls) that participated in the GWA study were genotyped using the allele-specific fluorescent primers (Table S2). The variant was analysed for association with PRA and compared with the most associated SNP markers, BICF2S23250878 and BICF2S23422025, using the software package PLINK [22].

The suggestive causative mutation for PRA3 in intron 4, *FAM161A_c_*_.1758-15_1758-16ins238_, was then screened in 247 TS. The panel of 247 TS (including the 80 DNA samples already sequenced), was made up of 35 PRA cases, 16 obligate carriers, 100 clear dogs and 96 dogs with unknown clinical status. In addition, samples from 99 dogs representing two breeds (23 LA and 76 TT) that are closely related to the TS breed were also included in the mutation screening.

The AHT Genetic Services department has tested 567 TS and 290 TT for the PRA3-associated variant over the course of 10 and six months respectively. These data were used to calculate mutant allele frequencies in the two breeds.

### Ethics statement

This study did not require ethics committee approval as DNA was collected using a relatively non-invasive procedure which did not require a license. Additionally, no live animals were involved in the research, nor were any in vivo experiments undertaken.

All samples were obtained from privately owned pet dogs with the owners' consent. The majority of DNA samples were obtained using non-invasive buccal/cheek swabs. Where DNA was obtained from blood, these samples were the remnants of blood drawn by veterinarians for routine and/or diagnostic veterinary purposes, and not specifically for the purposes of research. All eye examinations were conducted during the course of routine veterinary care and not specifically for research purposes.

## Supporting Information

Figure S1 **Genome-wide association mapping of PRA in Tibetan Spaniels.** -Log_10_ of p-values after correction for population stratification using the FMM approach. The red lines indicate the Bonferroni-corrected 5% significance level based on 15,674 SNPs. A prominent signal on CFA10 (p_fmm_ = 5.67×10^−6^) and a reduced inflation factor (λ = 1.27) was observed.
(PDF)

Figure S2 **Graphical comparison of the intron-exon boundaries of FAM161A.** A) Mouse (Mus muscularis) *FAM161A*. B) Human (Homo sapiens) *FAM161A*. C) Canine (Canis familiaris) *FAM161A* as predicted by Ensembl genebuild. Six genebuild exons (black) are identical to the mouse and human exons, while the intron-exon boundaries for five exons (grey) are inconsistent and four exons (white) bear little or no resemblance to human and mouse exons. D) Canine *FAM161A* exons confirmed by sequencing the retinal mRNA transcript. Exon 1 (red) was only partially sequenced. The location of the splice site insertion is indicated. E) Only the 3′-end of exon 1 (black) was successfully sequenced. Two alternative alleles were identified in retinal mRNA that differed from the CanFam2.0 reference sequence.
(PDF)

Figure S3 **Segregation of PRA in a large TS family.** A) Cases of PRA which have been genotyped at the PRA3 locus are highlighted. PRA3 (in red) and other forms of PRA (in yellow and green) tend to cluster separately for the most part, but there are cases were both segregate in the same families. The family of dogs shaded in gray can be rearranged to create the family in B (inset). B) The segregation of PRA3 is consistent with an autosomal recessive mode of inheritance. The PRA3 mutation (*FAM161A*_c.1758-15_1758-16ins238_) allele is represented by “−” and the wildtype allele by “+”. Clinical information pertaining to and DNA samples from 26 dogs was not available. Clinical information pertaining to a single dog (#21) that was homozygous for *FAM161A*_c.1758-15_1758-16ins238_ was unavailable.
(PDF)

Table S1 **Primers for microsatellite genotyping (Finemapping of TS_PRA locus on CFA10).**
(PDF)

Table S2 **Primers for sequencing and genotyping.**
(PDF)

Table S3 **Primers used for qPCR assays.** All probes were 5'-6FAM and 3'-Iowa black labelled, with internal ZEN labelling.
(PDF)
