# Supplementary figures and images for: An Intronic SINE Insertion in FAM161A that Causes Exon-Skipping Is Associated with Progressive Retinal Atrophy in Tibetan Spaniels and Tibetan Terriers
Source: PLoS One. 2014 Apr 4;9(4):e93990. doi: 10.1371/journal.pone.0093990 (PMC3976383; doi:10.1371/journal.pone.0093990)

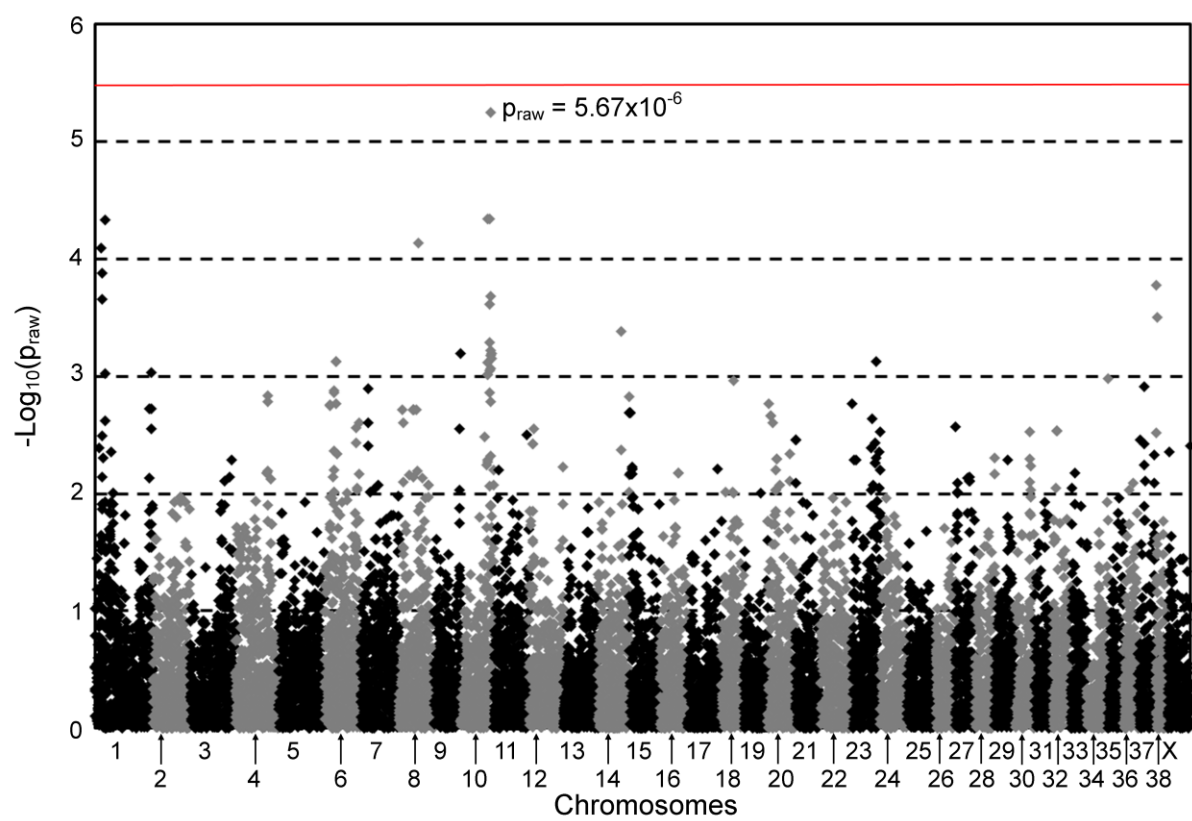

Supplement: Figure S1 — Genome-wide association mapping of PRA in Tibetan Spaniels. -Log10 of p-values after correction for population stratification using the FMM approach. The red lines indicate the Bonferroni-corrected 5% significance level based on 15,674 SNPs. A prominent signal on CFA10 (pfmm = 5.67×10^−6^) and a reduced inflation factor (λ = 1.27) was observed. (PDF) [file pone.0093990.s001.pdf]
