# Supplementary material for: An Intronic SINE Insertion in FAM161A that Causes Exon-Skipping Is Associated with Progressive Retinal Atrophy in Tibetan Spaniels and Tibetan Terriers
Source: PLoS One. 2014 Apr 4;9(4):e93990. doi: 10.1371/journal.pone.0093990 (PMC3976383; doi:10.1371/journal.pone.0093990)
Supplement: Figure S2 — Graphical comparison of the intron-exon boundaries of FAM161A. A) Mouse (Mus muscularis) FAM161A. B) Human (Homo sapiens) FAM161A. C) Canine (Canis familiaris) FAM161A as predicted by Ensembl genebuild. Six genebuild exons (black) are identical to the mouse and human exons, while the intron-exon boundaries for five exons (grey) are inconsistent and four exons (white) bear little or no resemblance to human and mouse exons. D) Canine FAM161A exons confirmed by sequencing the retinal mRNA transcript. Exon 1 (red) was only partially sequenced. The location of the splice site insertion is indicated. E) Only the 3′-end of exon 1 (black) was successfully sequenced. Two alternative alleles were identified in retinal mRNA that differed from the CanFam2.0 reference sequence. (PDF) [file pone.0093990.s002.pdf]

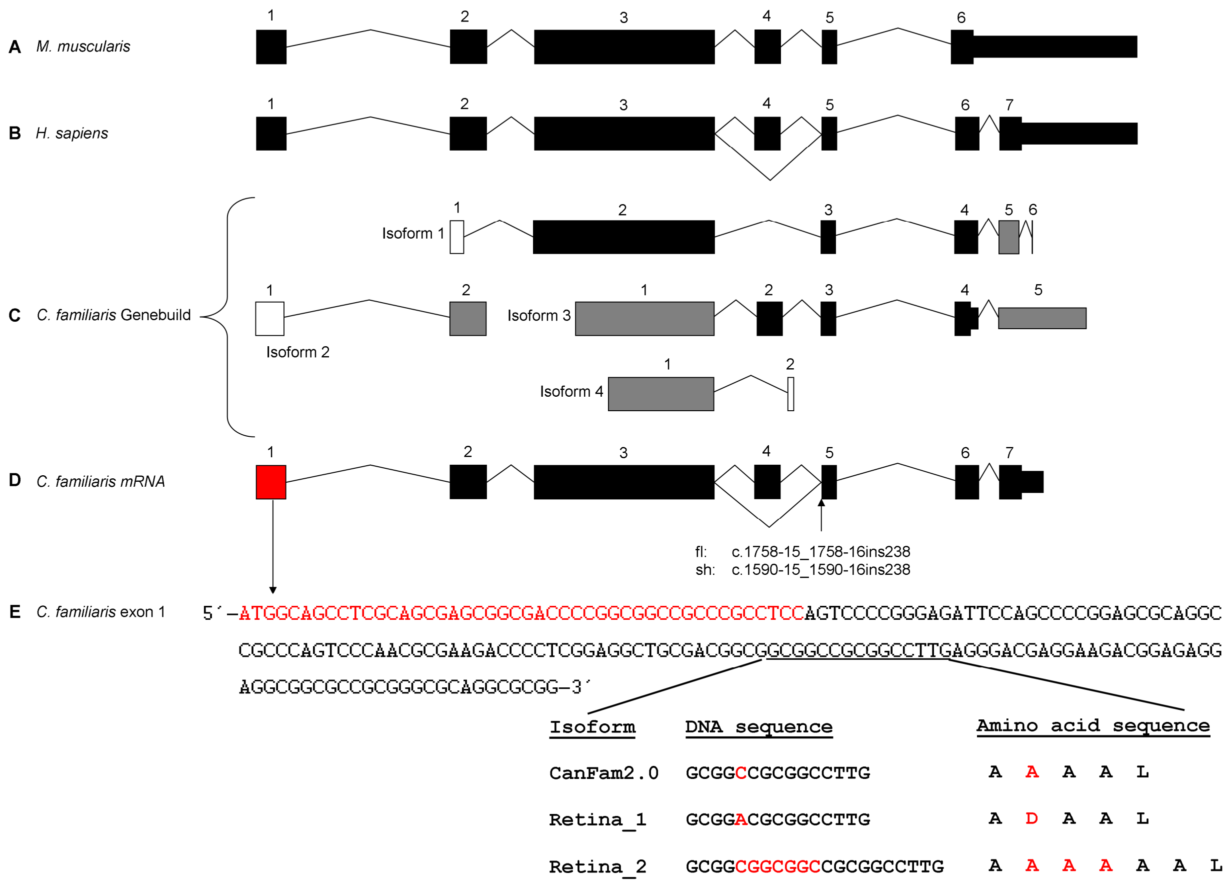

E) Canine retinal mRNA sequence differed from CanFam2.0 reference sequence in exon 1, and two variants were identified: the first was a SNP that changes an alanine residue to an aspartic acid residue; the second was a 6 bp insertion resulting in the in-frame insertion of two alanine residues. To assess the prevalence of these variants, genomic DNA from 43 TS and 76 dogs comprising 31 breeds was sequenced. The SNP variant was present in the homozygous and heterozygous state in both cohorts (i.e. TS and multiple other breeds). Eight dogs from five additional breeds (Australian Shepherd, Brittany Spaniel, Shetland Sheepdog, Tibetan Terrier and Welsh Springer Spaniel) were heterozygous for the insertion variant and none were homozygous. It is unclear what effect, if any, these variants have on the protein.
