# Supplementary material for: An Intronic SINE Insertion in FAM161A that Causes Exon-Skipping Is Associated with Progressive Retinal Atrophy in Tibetan Spaniels and Tibetan Terriers
Source: PLoS One. 2014 Apr 4;9(4):e93990. doi: 10.1371/journal.pone.0093990 (PMC3976383; doi:10.1371/journal.pone.0093990)
Supplement: Figure S3 — Segregation of PRA in a large TS family. A) Cases of PRA which have been genotyped at the PRA3 locus are highlighted. PRA3 (in red) and other forms of PRA (in yellow and green) tend to cluster separately for the most part, but there are cases were both segregate in the same families. The family of dogs shaded in gray can be rearranged to create the family in B (inset). B) The segregation of PRA3 is consistent with an autosomal recessive mode of inheritance. The PRA3 mutation (FAM161A c.1758-15_1758-16ins238) allele is represented by “−” and the wildtype allele by “+”. Clinical information pertaining to and DNA samples from 26 dogs was not available. Clinical information pertaining to a single dog (#21) that was homozygous for FAM161A c.1758-15_1758-16ins238 was unavailable. (PDF) [file pone.0093990.s003.pdf]

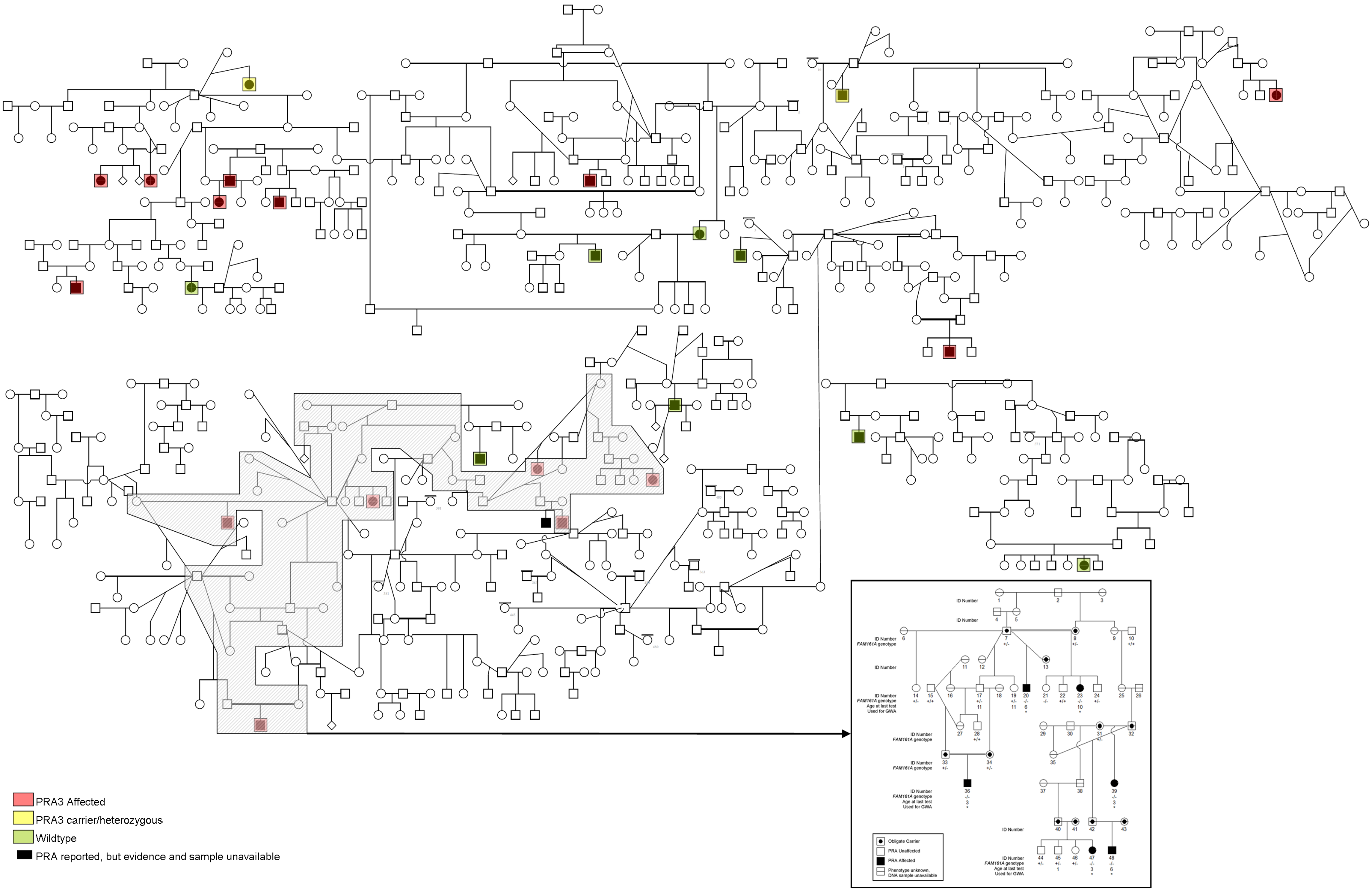

ID Number
FAM161A genotype
Age at last test
Used for GWA
Obligate Carrier
PRA Unaffected
PRA Affected
Phenotype unknown, DNA sample unavailable
PRA3 Affected
PRA3 carrier/heterozygous
Wildtype
PRA reported, but evidence and sample unavailable
