# Supplementary material for: An Intronic SINE Insertion in FAM161A that Causes Exon-Skipping Is Associated with Progressive Retinal Atrophy in Tibetan Spaniels and Tibetan Terriers
Source: PLoS One. 2014 Apr 4;9(4):e93990. doi: 10.1371/journal.pone.0093990 (PMC3976383; doi:10.1371/journal.pone.0093990)
Supplement: Table S1 — Primers for microsatellite genotyping (Finemapping of TS_PRA locus on CFA10). (PDF) [file pone.0093990.s004.pdf]

F primer Name	F primer Seq (5'-3') (TAIL = TGACCGGCAGCAAAATTG)	R primer Name	R primer Seq (5'-3')	Product size (bp)	Tm	Genomic location (bp)	Microsatellite
CFA10_61.66F	TAIL-TAGACCCAGGCAATTCAAATC	CFA10_61.66R	TTGAGGATATCAAACCCTTCAC	193	56	61,659,255	(CA)14
CFA10_61.75F	TAIL-ATGGAACTGCCATATGATCCA	CFA10_61.75R	GCATGTCCCTTGTTATGGCTA	277	56	61,752,065	(CA)21
CFA10_61.87F	TAIL-AGCTGGGTCCCTTTCAGTTTA	CFA10_61.87R	CAGCCAGTACGTCTGATGCTT	236	56	61,868,609	(GT)20
CFA10_61.96F	TAIL-CCTTTGTGCATTCTCTGTTCC	CFA10_61.96R	TCCCTAGGCCTACAGGATTT	234	56	61,963,812	(GT)15
CFA10_62.34F	TAIL-TCTGCTCTTTAAGGCCTGATG	CFA10_62.34R	GAATGCACTTCAACCACCATT	212	56	62,337,915	(GT)18
CFA10_62.43F	TAIL-TCTGTTCAAGAGCTGTGTGGAT	CFA10_62.43R	TTCTGTGGCACTGCTTCCTT	274	56	62,434,740	(GT)15
CFA10_62.49F	TAIL-ACTGAGGGATGCAGAAGTGAA	CFA10_62.49R	ATATCTGAGCGCAGCATGAAG	174	56	62,487,468	(CA)16
CFA10_62.55F	TAIL-TTTAAGAGCCAACCACTTCCA	CFA10_62.55R	TGCTTCTTCACCCAGATTGTT	229	56	62,552,700	(CA)20
CFA10_62.79F	TAIL-GCCAAATTATGCAAGTAGACCA	CFA10_62.79R	GCATGATACCCCTTTAGATTCACC	176	56	62,786,640	(GT)15
CFA10_62.98F	TAIL-CCTGCATGTTCTCACAAAGGT	CFA10_62.98R	GTGGTGGTAGTTGGACAATGC	219	56	62,978,760	(CA)17
CFA10_63.01F	TAIL-ACCTCACTGCAGGCTCACTT	CFA10_63.01R	TGAATGGAAGCTGTGTTTGTTC	247	56	63,008,220	(CT)21
CFA10_63.20F	TAIL-ATCACCTCAAATCCACCACAA	CFA10_63.20R	TATTCCCTTGGGCAGGTTTAC	241	56	63,199,200	(CA)15
CFA10_63.33F	TAIL-ATGAAAGCAAAGCAGGGAGAT	CFA10_63.33R	ATTGAAGGCTGAGGGAGCTAA	209	56	63,331,680	(CA)17
CFA10_63.40F	TAIL-TAGCACCAGAACCTTCCACTC	CFA10_63.40R	GCATCACAACACTCACTCTGC	205	56	63,402,960	(CA)21
CFA10_63.51F	TAIL-GGAGTTTACCATAAGTAAGCTTCTCC	CFA10_63.51R	TGCTTCTGACCATTCCACAAT	228	56	63,509,760	(CA)18
CFA10_63.71F	TAIL-TCCTCTCCTCCAAACACTTACC	CFA10_63.71R	ATGCATACAGCACTTGCACAC	184	56	63,705,840	(GT)16
CFA10_63.82F	TAIL-CGCAGCCTTTAAATCATCCTT	CFA10_63.82R	GCAGCTCACAATGAACCATTT	249	56	63,820,800	(GT)17
CFA10_63.86F	TAIL-TTCCATTCTGAAGGCCCTAAT	CFA10_63.86R	AAGGTGTGCTGAAAGGAAAGC	244	56	63,859,200	(GT)22
CFA10_63.91F	TAIL-CTCCACCACTTGCTCTCTCAT	CFA10_63.91R	GAACCCTATGAAGCAACAGCA	205	56	63,913,440	(CT)17
CFA10_63.93F	TAIL-CCCTACCCAAGTTCTACCCTTT	CFA10_63.93R	TTCAGCATGACAAGGGACAC	242	56	63,934,627	(GA)16
CFA10_64.00F	TAIL-GCAGCACTATCAATAGCCAAA	CFA10_64.00R	TCTACTTCATCCATGTCCTTGC	177	58	63,998,363	(GT)19
CFA10_64.08F	TAIL-CCCTGTATTGATTAGAGCACGA	CFA10_64.08R	GATCACCTTGGTTTCTGGTCA	235	56	64,075,600	(CA)19
CFA10_64.112F	TAIL-GCATCTGCAATCAATTTGAGG	CFA10_64.112R	CTCCCTCTCCATTTGTCCTCT	265	58	64,112,403	(AT)17
CFA10_64.113F	TAIL-CATCAGTTCATCCCGTAGCAT	CFA10_64.113R	GGCACAGTAGGAGATTAACAGCA	194	56	64,113,386	(GA)14
CFA10_64.19F	TAIL-ACTGCACCCATGTCTTGTTCT	CFA10_64.19R	GGTGTTCAGTATGTGGCTATGTTC	237	56	64,186,104	(GT)14
CFA10_64.20F	TAIL-AAGTTCATGCCAATATCCTAGAGTC	CFA10_64.20R	TTGTTGTTGCCTATTGGGTTC	366	56	64,201,234	(GA)16
CFA10_64.22F	TAIL-GCAGTTGAATAACCTGCTTGC	CFA10_64.22R	TATCCTTTCTTCCTGCCACCT	246	56	64,217,353	(CA)16
CFA10_64.51F	TAIL-AGACCAGCTTTTTGCTTCAGA	CFA10_64.51R	CTGCGATTAAATGGTCAAAGG	267	57	64,508,603	(TG)14
CFA10_64.57F	TAIL-CATGTTGTCATGGAATGCAAG	CFA10_64.57R	AGTGAACCCCGACAGGTTAGT	309	57	64,572,573	(AC)11
CFA10_64.71F	TAIL-CCCCTAAGCCTGATCCAATAA	CFA10_64.71R	TGAAAGGAAATGAGTAGTTTTGCT	275	57	64,713,419	(TG)12
CFA10_65.18F	TAIL-CAAGGGTCTAATTTCCCTTTCC	CFA10_65.18R	TGGGTACCAAGTGGAATTTTG	127	57	65,182,153	(AC)18
CFA10_65.44F	TAIL-TGAACTCCTCAGACCTGCAAT	CFA10_65.44R	CTCTGCTCCAGTCAGATTTGG	304	57	65,447,202	(AG)16
CFA10_65.53F	TAIL-AGCAGTTTTGAAGCATCATGG	CFA10_65.53R	AAAAGTAGCCCAAGATCAACCA	120	57	65,529,249	(TG)18
CFA10_65.63F	TAIL-CAGATCTTGAGAGGGAAAGAGAA	CFA10_65.63R	CATAGTTTCTTGCCAAGTACTGTCT	220	56	65,625,939	(TA)20
CFA10_65.78F	TAIL-GTTCACCTCCACTCCCATTTA	CFA10_65.78R	GGCACAGAACAAAACATCCAT	314	57	65,784,034	(TC)14
CFA10_65.93F	TAIL-CCTGTTGTGTTCAGCAAGGAT	CFA10_65.93R	AGTTATCGCTGCCTTCTCCTC	358	57	65,931,460	(TG)11(TA)10
CFA10_66.01F	TAIL-TGCAAAAGTGAGAGACAGATCC	CFA10_66.01R	TGCTGTGTTATCTGCAGCTGTT	322	57	66,013,662	(GT)12
CFA10_66.06F	TAIL-CCGTATTCCTCCCTGTAGTCC	CFA10_66.06R	TCCAGATTTCACAGCATGATTC	317	57	66,058,427	(AT)20
CFA10_66.16F	TAIL-CTCTGTGTTTATTGGCGCATT	CFA10_66.16R	GGCGAGACCTCATTCTTTTCT	183	56	66,162,019	(AC)15
CFA10_66.3F	TAIL-GCAGACAGCCTTTTGTTTGAC	CFA10_66.3R	AGTTCTACTTTGCCCGGCTAC	202	60	66,303,329	(GT)14
CFA10_66.38F	TAIL-GGGAAGAAAGAAACTCTTGGAA	CFA10_66.38R	TCCCTTCTTTCCATTACCTCTG	269	57	66,379,163	(CA)11
CFA10_66.4F	TAIL-CCCCACACATCAACATAGGTC	CFA10_66.4R	GCTATGCAGGCATTTATTTTGG	296	57	66,395,178	(TA)14
CFA10_66.45F	TAIL-GTGAAATGTATTCCGCATGGT	CFA10_66.45R	ATGTCCTATGGCCTTTTGGTT	242	57	66,448,543	(TA)18
CFA10_66.51F	TAIL-GGAGAAGAGGAAGTTTTGCAG	CFA10_66.51R	GCCAAAGTCTCAAAGGGATCT	314	56	66,509,954	(TC)14
CFA10_66.56F	TAIL-CACTGGCACCAGGTTAAATGT	CFA10_66.56R	CCTATGGGGCTCTGGATAAAT	348	57	66,557,299	(AG)12
CFA10_66.61F	TAIL-CTCCTACTGGGAGCCAAAAAC	CFA10_66.61R	CCACATCCTTCCTACCACTGA	212	57	66,611,818	(AC)20
CFA10_66.68F	TAIL-GCTGCATTGCTGCTTATTTTC	CFA10_66.68R	AGGTTTAAGGCAGCACCAATC	296	57	66,684,676	(TC)21(TG)20
CFA10_66.71F	TAIL-CCACCCTCATGTTCATCTGTT	CFA10_66.71R	CACCATCACTCACCAATAGGG	301	57	66,710,025	(TG)16
CFA10_66.99F	TAIL-TTAGGATTGTGGGGTTCAGTG	CFA10_66.99R	TCAGGTGAAAGGAAGAAGCAA	304	57	66,985,774	(CT)15
CFA10_67.06F	TAIL-GAAAGTGGAGGGGAAAGTGAC	CFA10_67.06R	AACCGGGTTGAACCATTTTT	236	57	67,056,476	(AC)15
CFA10_67.16F	TAIL-CTGCACTGTTTCCTTCCTCTG	CFA10_67.16R	ATCTGGGGTCTTTTTGCTGTT	270	57	67,164,626	(CA)21
CFA10_67.39F	TAIL-CCAGGTTTGGGGTGTATTTCT	CFA10_67.39R	CCAGTGGTCACCTCAACAAGT	244	57	67,385,860	(GT)13
CFA10_67.48F	TAIL-TGAACCTCCGAAGTCACTTGT	CFA10_67.48R	GGAAAAGTGTAAAGGGGACCA	258	57	67,482,874	(TC)19
CFA10_67.73F	TAIL-TGCTTCTGAGGCACGAGTATT	CFA10_67.73R	TGCTTGCGTTGCTATTAAGTTC	302	57	67,729,134	(GA)13
CFA10_67.94F	TAIL-GGTTCATGCCCTCTAATAGGC	CFA10_67.94R	ATGCCCTCACACTGAAATCAC	237	57	67,940,561	(GT)10
CFA10_68.04F	TAIL-CTCCGGGGACTCTCAAATTAC	CFA10_68.04R	TCTGACTTGGGAAAGTGGTTG	263	57	68,043,943	(AC)20
CFA10_68.11F	TAIL-TCTAGGCTGGGCTTTGAGAAT	CFA10_68.11R	TGTCAATTGATCCCACTCCAT	277	57	68,110,195	(GT)32
CFA10_68.42F	TAIL-TTTCCTTGCTCCATTGATGTC	CFA10_68.42R	CAGAATGTGGGTGCTTTTCAT	314	57	68,422,023	(GT)17
CFA10_68.54F	TAIL-GTGCACCATCTGGACTACCAT	CFA10_68.54R	ACAGTGCATTACAGGGACCAG	199	57	68,544,337	(TC)24
CFA10_68.61F	TAIL-AAAACCTTTCTCCTCCCCTTC	CFA10_68.61R	ATGTAGGCCCACTTTTTGCTT	184	57	68,607,662	(CA)15

PCR reaction components	Final Concentration
dNTPs (NEB)	0.2 mM
Tailed Forward primer	0.1 μM
Reverse Primer	0.25 μM
5'-6FAM-CAATTTTGCTGCCGGTCA-3'	0.1 μM
PCR buffer (Applied Biosystems)	1X
$MgCl_2$ (Applied Biosystems)	1.5 mM
AmpliTaq Gold DNA Polymerase (Applied Biosystems)	1.2 units
DNA template	40 ng
PCR grade water	up to 12 μL

Thermal Cycling Parameters	
94℃ - 4 min	
94℃ - 1 min	X30
Tm℃ - 1 min	
72℃ - 2 min	
94℃ - 1 min	X8
50℃ - 1 min	
72℃ - 2 min	
72℃ - 30 min	
