# Supplementary material for: An Intronic SINE Insertion in FAM161A that Causes Exon-Skipping Is Associated with Progressive Retinal Atrophy in Tibetan Spaniels and Tibetan Terriers
Source: PLoS One. 2014 Apr 4;9(4):e93990. doi: 10.1371/journal.pone.0093990 (PMC3976383; doi:10.1371/journal.pone.0093990)
Supplement: Table S2 — Primers for sequencing and genotyping. (PDF) [file pone.0093990.s005.pdf]

F primer Name	F primer Seq (5'-3')	R primer Name	R primer Seq (5'-3')	Product size (bp)
**Primers for sanger sequencing of retinal cDNA**				
FAM161A_cDNA1_F	CATGGCAGCCTCGCAG	FAM161A_cDNA1_R	AAGTTGGGCAACTCATCTTCA	540
FAM161A_cDNA2_F	CCACTTGGAAACTATGGCAAA	FAM161A_cDNA2_R	TTTGCACTTGCACTTCTTCCT	1203
FAM161A_cDNA3_F	TTTTCCCCCTTTATCATGACC	FAM161A_cDNA3_R	CCAGGCTACAGATGACTTTGC	1423
FAM161A_cDNA4_F	GCAGACATTGCAGCAGATGAG	FAM161A_cDNA4_R	CTTTGAAACAAACTCATCAG	510
**Primers for sanger sequencing of gDNA**				
FAM161A_ex5_F	TTCATCTCTTCAGGCCAGTGT	FAM161A_ex5_R	ACTGCAGTAGCAGCGTGTTCT	720
**Allele-specific fluorescent primers for genotyping of SINE insertion**				
FAM161A_AFF_F	GGATCCCTTTATTTGATTTTAGAAAG	FAM161A_R	6FAM-CAACAAACACAACCTGAGCAA	137
FAM161A_NOR_F	TCCCTTCCTTTTATTTGATTTTAGAAAG	FAM161A_R	6FAM-CAACAAACACAACCTGAGCAA	135

PCR amplification for Sanger sequencing of gDNA and cDNA

PCR reaction components	Final Concentration
dNTPs (NEB)	0.2 mM
Forward primer	0.5 µM
Reverse Primer	0.5 µM
PCR buffer (Qiagen)	1X
HotStarTaq Plus DNA Polymerase (Qiagen)	1.2 units
Q solution - optional (Qiagen)	1X
DNA template	40 ng
PCR grade water	up to 12 µL

Thermal Cycling Parameters

Thermal Cycling Parameters	
94℃ - 10 min	
94℃ - 1 min	X35
57℃ - 1 min	
72℃ - 2 min	
72℃ - 10 min	

PCR amplification for genotyping of SINE insertion

PCR reaction components	Final Concentration
dNTPs (NEB)	0.2 mM
FAM161A_AFF_F	0.08 µM
FAM161A_NOR_F	0.08 µM
FAM161A_R	0.08 µM
PCR buffer (Qiagen)	1X
HotStarTaq Plus DNA Polymerase (Qiagen)	0.6 units
DNA template	40 ng
PCR grade water	up to 12 µL

Thermal Cycling Parameters	
94℃ - 10 min	
94℃ - 1 min	X35
60.5℃ - 1 min	
72℃ - 2 min	
72℃ - 10 min	
