# Supplementary material for: An Intronic SINE Insertion in FAM161A that Causes Exon-Skipping Is Associated with Progressive Retinal Atrophy in Tibetan Spaniels and Tibetan Terriers
Source: PLoS One. 2014 Apr 4;9(4):e93990. doi: 10.1371/journal.pone.0093990 (PMC3976383; doi:10.1371/journal.pone.0093990)
Supplement: Table S3 — Primers used for qPCR assays. All probes were 5'-6FAM and 3'-Iowa black labelled, with internal ZEN labelling. (PDF) [file pone.0093990.s006.pdf]

Assay Name	F primer Seq (5'-3')	Probe sequence (5'-3')	R primer Seq (5'-3')	Product size (bp)	Efficiency (%)	r2
FAM161A_FL	TGCAGAAACTCCTGACAATCC	GGGCTAAAGCTTATGACTCCCATCAAA	TTCTCTCATCCTTTCCTTTTCAC	116	102.5	0.9904
FAM161A_FL-5	TGCAGAAACTCCTGACAATCC	GGGCTAAAGCTTATGACTCCCATCAAA	GCTTTTCTGCTGCCATTCTT	116	104.9	0.9924
FAM161A_SH	GGGAGAGAACAAGCCACAAG	GGCCGCTACTGTTTGAAAGAGTTGC	GCTGCCATTCTTGCATTTTT	140	102.9	0.9923
FAM161A_SH-5	AGCCTGTGCCTTGTAACTGC	CCCCCTCCAATGCCCACAGT	GCTGCCATTCTTGCATTTTT	90	106.4	0.9938
TBP	TCTGGCATATTTCCTCGCTG	ACTGTTCTTCACTCTTGGCTCCCG	TTCAGTTCTGGGAAGATGGTG	90	106.8	0.9938
ACTB	CCAACCGTGAGAAGATGACC	CGAGACTTTCAACACCCCAGCCA	CGTACAGGGACAGCACAG	90	97.9	0.995

PCR reaction components	Final Concentration
KAPA Probe Fast Universal qPCR mastermix (KAPA Biosystems)	1X
PrimeTime qPCR assay mix (IDT)	1X
cDNA	4 μL
PCR grade water	up to 20 μL

Thermal Cycling Parameters	
95℃ - 5 min	
95℃ - 10 sec<br>57℃ - 15 sec<br>72℃ - 15 sec	X40
